# Maternal Education and Micro-Geographic Disparities in Nutritional Status among School-Aged Children in Rural Northwestern China

**DOI:** 10.1371/journal.pone.0082500

**Published:** 2013-12-10

**Authors:** Cuili Wang, Robert L. Kane, Dongjuan Xu, Lingui Li, Weihua Guan, Hui Li, Qingyue Meng

**Affiliations:** 1 Shandong University School of Nursing, Jinan, Shandong, China; 2 Shandong University Center for Health Management and Policy, Jinan, Shandong, China; 3 University of Minnesota School of Public Health, Minneapolis, Minnesota, United States of America; 4 Ningxia Medical University College of Management, Yinchuan, Ningxia, China; 5 Peking University China Center for Health Development Studies, Beijing, China; University of Alabama at Birmingham, United States of America

## Abstract

**Objectives:**

Prior evidence suggests geographic disparities in the effect of maternal education on child nutritional status between countries, between regions and between urban and rural areas. We postulated its effect would also vary by micro-geographic locations (indicated by mountain areas, plain areas and the edge areas) in a Chinese minority area.

**Methods:**

A cross-sectional study was conducted with a multistage random sample of 1474 school children aged 5-12 years in Guyuan, China. Child nutritional status was measured by height-for-age z scores (HAZ). Linear mixed models were used to examine its association with place of residence and maternal education.

**Results:**

Micro-geographic disparities in child nutritional status and the level of socioeconomic composition were found. Children living in mountain areas had poorer nutritional status, even after adjusting for demographic (plain versus mountain, *β* = 0.16, *P* = 0.033; edge versus mountain, *β* = 0.29, *P* = 0.002) and socioeconomic factors (plain versus mountain, *β* = 0.12, *P* = 0.137; edge versus mountain, *β* = 0.25, *P* = 0.009). The disparities significantly widened with increasing years of mothers’ schooling (maternal education*plain versus mountain: *β* = 0.06, *P* = 0.007; maternal education*edge versus mountain: *β* = 0.07, *P* = 0.005). Moreover, the association between maternal education and child nutrition was negative (*β* = -0.03, *P* = 0.056) in mountain areas but positive in plain areas (*β* = 0.02, *P* = 0.094) or in the edge areas (*β* = 0.04, *P* = 0.055).

**Conclusions:**

Micro-geographic disparities in child nutritional status increase with increasing level of maternal education and the effect of maternal education varies by micro-geographic locations, which exacerbates child health inequity. Educating rural girls alone is not sufficient; improving unfavorable conditions in mountain areas might make such investments more effective in promoting child health. Nutrition programs targeting to the least educated groups in plain and in edge areas would be critical to their cost-effectiveness.

## Introduction

Child linear growth as measured by height for age Z score (HAZ) is internationally recognized as a robust indicator of nutritional and health status [1], [2]. Stunting, linear growth failure (low HAZ), indicates chronic under-nutrition and reflects the cumulative effects of under-nutrition, recurrent infections, inadequate child care and poor socioeconomic environments. The negative ramifications of stunting can extend from childhood to adulthood, with higher morbidity and mortality, delayed cognitive development, poor educational achievement, short stature, reduced work capacity, and poor reproductive performance [3]–[6]. Child malnutrition is currently the leading cause of the global burden of disease and ending malnutrition is an agenda of the current millennium, particularly for the developing world [5].

A considerable body of research suggests that maternal education is the most important factor in explaining differentials in child health outcomes, more important than paternal education, health service availability, and family income [7]–[11]. A positive association with height or stunting has been found in many countries [1], [10], [12]–[20]. More educated mothers presumably might more efficiently use health resources and thus improve the effectiveness of child health services [13], [21]. They also might tend to marry more socioeconomic advantaged men and be employed in the market, thereby increasing family resources [22]. Moreover they might be more concerned about and preferably invest in child nutrition [21]–[23]. However, the effect is not consistent. Some studies found insignificant relationship [24], while a few found negative association [25]. An insignificant effect was found in a study in Bangladesh, explained by low social status of women and their limited health decision-making power [24]. A negative effect at the lower level of maternal schooling was shown in a study from Ghana, reflecting the threshold effect of education [25]. Additionally, there is extensive research for under-five children compared to a paucity of data for school children [6]. Further insight is needed into the relation between maternal education and school child malnutrition.

The degree to which geographic locations may affect the association of maternal education with child malnutrition is of interest. Child nutritional status has been shown to be worse in less-developed countries, regions or rural areas compared to their counterparts [7], [10], [13]. A study using the major nutritional surveillance systems in Indonesia and in Bangladesh showed that the effect of maternal education on child stunting was the same in Indonesia as that in Bangladesh but there were significant urban-rural differentials in the size of its positive effect in Indonesia, where maternal education mattered more in rural areas, but not in Bangladesh [10]. Another study using Demographic and Health Survey data from 36 developing countries in three regions of South Asia (SA), Sub-Saharan Africa (SSA) and Latin America and the Caribbean (LAC) showed that association of maternal education with child HAZ appeared to be weaker in SSA and their associations in rural areas of SA and of LAC were stronger than in urban areas but not in SSA [7]. Moreover, the magnitudes of the urban-rural differences found in both studies were not large. Generally, the previous studies regarding geographic variations in the effect of maternal education on child malnutrition were mainly undertaken at the broad level of the developing country regions (e.g. countries and regions within them). However, the conclusions reached might differ for more localized areas, due to the unique aspects and circumstances that characterize life in specific or target population.

Similarly, there are still significant geographic variations in child malnutrition between urban and rural areas and between regions in China [26]–[28]. Prevalence of rural malnutrition in under 5 years old children tripled that in the cities in 2007 [26]. A field survey from rural poor areas in China in 2000 showed that the prevalence of stunting among 733 children under 18 years old in the total sample was 43%, nearly double the average prevalence for the whole rural China (22.6%), and the rate in a subsample of minority areas was even higher (72%) [29]. Although extensive studies on the relationship between maternal education and child malnutrition have been implemented internationally, it has not been systematically explored in China. The few Chinese studies found the mixed results but generally positive effect [29]–[31]. One study with a negative effect on HAZ in poor areas explained that educated mothers were more likely to be employed in other jobs, except farming and thus having less time to spend on care of children [29]. Another study using the China Health and Nutrition Survey (CHNS) data in 2000 indicated that child HAZ was not significantly improved by maternal education but mainly by increased household income and decreased family size [27]. Additionally, to our knowledge, no Chinese research has yet linked the effect of maternal education on child malnutrition with geographic variations.

Better information about the relationship of maternal education with nutritional status among school-aged children in more localized areas would provide a platform for cost-effective policies and programs for the target population. There is evidence of inconsistent associations between maternal education and child malnutrition across geographic areas. The Ningxia School-aged Children Health Project, implemented in Guyuan, Ningxia Hui Autonomous Region, aimed to improve the health status of such vulnerable population of rural school-aged children in the poor minority area of Northwestern China. Using the survey data, we pose the following questions: (1) Are there micro-geographic variations in child chronic malnutrition between place of residence (classified as mountain areas, plain areas and the edge between mountain and plain areas, where mountain areas represent the relatively unfavorable environments)? (2) How does the association between maternal education and child malnutrition vary by place of residence? Thus, we hypothesize that children in mountain areas have worse nutritional status than their counterparts and the association of maternal education with child malnutrition in mountain areas may differ from that in plain areas and in the edge between mountain and plain areas, respectively.

## Materials and Methods

### Study setting

This is a population-based cross-sectional study among rural elementary school children conducted in June 2010 in Guyuan City, a prefecture-level city of Ningxia Hui Autonomous Region. Ningxia is an underdeveloped region in Northwestern China. Guyuan is the poorest area in China; the disposable income per capita of rural residents was 2962 yuan (434US$) compared to 5153 yuan (754US$) for the whole country in 2009. The population of Guyuan was about 1.5 million, 45.3% of which were Hui minorities. The educational level of the region, with an average of six years of schooling among rural and urban women aged 18 and older, is far lower than the national level and eastern level. Guyuan is located at the northwest edge of the Loess Plateau and in the Liupanshan mountain area of Southern Ningxia. In 1972, the United Nations declared this region unsuitable for human survival. Even here, there is substantial micro-geographic variation in living situations.

### Sampling and data collection

A multistage random sample of 1474 children aged 5–12 years in seven elementary schools of Guyuan was enrolled. First, of the four counties and one district in Guyuan, two counties (Xiji and Pengyang) and one district (Yuanzhou) were included in this survey. Seven township elementary schools from three counties or districts were randomly selected from a list stratified by county and, within the county, by economic status and school size. All students (Grade 1–6) in each selected school were studied. The sampled schools were visited on pre-arranged dates in June 2010. Thirty-three trained medical undergraduates and nine postgraduates, led by the principal investigators, collected the data. It took one week to complete data collection. The anthropometric parameters of the selected children were measured. Analogue physician health scales were used to measure height (China Jiangsu Wuxi Weighing Apparatus Factory). All the instruments were standardized before the measurements and balances were zero calibrated on a daily basis. This was done with the child standing erect without shoes and with the eyes looking horizontally and the feet together on a horizontal level. These measurements were done to the nearest 0.1 cm. The measurement variations between investigators during the training were less than 0.5 cm, so were those between different scales.

The study instruments included two sets of structured questionnaires. One is administered for children, and another for the children’s family. The questionnaire for children includes data on socio-demographics, relationships with peers, teachers and parents, and health status; another for family consists of data on socio-demographics, family structure, community environment, and health status. The data in this study were mainly derived from the questionnaire for family. Children’s guardians, of whom mother was preferred, were interviewed face-to-face in schools or in village clinics. Children completed the questionnaires with the guidance of investigators in the presence of their teachers in their classrooms. Finally, the study contained observations for 1474 children from 1116 households. Quality control measures and good practices included training of data collection team, pre-testing of processes and materials and field monitoring of data collection. Timely availability of the study instruments, meeting of data collection team at the end of everyday to share experiences and submit completed forms, and trouble-shooting field problems were ensured.

### Definitions of variables

**Child HAZ.** Child height-for-age z score (HAZ) was generated by using WHO 2007 reference values [32] and comparable across different ages and genders. HAZ measure is accepted as a particularly good and robust indicator of overall child health as it is a measure of long-term or chronic malnourishment and doesn’t fluctuate with short-term malnutrition [1], [2]. Stunting is defined as HAZ of less than minus two standard deviation (-2 SD) of the mean of WHO reference 2007 [32].

**Maternal education and place of residence.** Maternal education was indicated by formal years of schooling that mothers had attained. Place of residence was a trichotomized micro-geographic location variable (1, plain areas; 2, the edge between mountain and plain areas; versus 0, mountain areas). Mountain areas are under such harsh natural and living environment, deficient in good soil and water resources that are helpful for crop growth and residents living. By contrast, plain and the edge areas are relatively rich in such resources.

**Potential confounders.** The following covariates served as control variables in multivariate analyses: individual characteristics including child age in years, child gender (1, boy; versus 0, girl), child ethnicity (1, Han majority; versus 0, Hui minority), household socioeconomic composition including paternal education (formal years of schooling), household income indicated by logarithm of the household disposable income per capita in 2009, resource of drinking water (1, piped water; versus 0, non-piped water including well water, river water or cellar water gathering rainfall), time access to health services indicated by logarithm of time access from household to the nearest health clinics, maternal age in years, and household size.

### Statistical analyses

Participant characteristics were compared across category of place of residence using *χ^2^* or ANOVA tests for categorical and continuous variables, respectively. Linear mixed models (random intercept models) were applied to examine whether there are micro-geographic disparities in child HAZ and whether the association of maternal education with child HAZ may vary by place of residence. This model allows adjustment for sibling cluster effects within household (i.e. heteroskedasticity) by disentangling variance of HAZ occurring at household and individual levels. The multivariate fixed effect model was constructed based on the reduced-form household health production function and the framework of external determinants of child health [22], [23], [33]. First, to examine whether there were micro-geographic disparities in child nutritional status, place of residence and demographic characteristics of children were entered into the model. Second, to examine whether micro-geographic disparities were mitigated by socioeconomic composition, socioeconomic factors were added into the first model on parental education, household income, resource of drinking water, access to health clinics, and as well as maternal age and household size. Last, the product term with maternal education and place of residence was entered into the second model to test whether association of maternal education with child HAZ may vary by place of residence. Post-hoc estimation using *Wald z* tests were conducted on coefficients for maternal education on child HAZ in plain areas and in the edge areas. The collinearity diagnosis showed no multicollinearities in independent variables. Analyses were conducted using Stata v.12 (StataCorp, College Station, TX).

### Ethics statement

Written informed consent forms were used to get approvals from participants on the collection and use of the data. Written informed consent forms were also obtained from guardians on the behalf of children participants involved in the study. The study was approved by the Institutional Review Board of Shandong University.

## Results

### Descriptive statistics

Table 1 summarizes the descriptive statistics for the total sample and subsamples stratified by place of residence. The mean child HAZ was significantly lower compared with WHO child growth standards (*t* = –24.93, *P*<0.001); the prevalence of stunting was 11.68% in the total sample. The proportion of illiterate mothers was 45.93%; the average years of schooling mothers attained was only 3.37. Fathers had higher levels of education than mothers. The median household income per capita was 3000 yuan (441US$). About 30% of children resided in mountain areas, 50% in plain areas and 20% in the edge between mountain and plain areas. Only 25% of children used piped water; the median time from household to the nearest clinics was 10 minutes.

**Table 1:** Descriptive summary^a^ of child nutritional status and demographic, socioeconomic factors.

	Total sample(n = 1474)	Place of residence		
		Plain areas (n = 764)	Edge areas (n = 298)	Mountain areas (n = 412)
Child HAZ^b^	–0.78 (1.19)	–0.74 (1.10)	–0.59 (1.17)	–0.96 (1.34)***
Stunting	11.68%	10.66%	8.19%	16.06%**
Maternal education	3.37 (3.84)	3.66 (3.86)	3.98 (3.83)	2.39 (3.64)***
Maternal illiterate rate^c^	45.93%	40.80%	38.33%	60.64%***
Child age	10.45 (2.05)	10.41 (1.97)	10.38 (2.19)	10.59 (2.09)
Child gender: girls	50.27%	50.39%	47.62%	52.18%
Child ethnicity: Hui	55.22%	51.05%	48.98%	67.72%***
Paternal education	5.74 (3.97)	6.01 (3.81)	6.02 (3.65)	5.05 (4.39)***
Paternal illiterate rate^c^	19.01%	17.04%	14.14%	26.29%***
Log household income per capita	3.45 (0.42)	3.48 (0.42)	3.42 (0.42)	3.42 (0.40)
Household size	5.24 (1.38)	5.24 (1.46)	5.33 (1.32)	5.19 (1.25)
Maternal age	35.14 (4.56)	35.21 (4.61)	35.18 (4.28)	34.98 (4.66)
Non-piped water	74.80%	70.38%	78.16%	81.29%***
Log time access to nearest clinics	1.00 (0.38)	0.94 (0.35)	1.01 (0.38)	1.10 (0.41)***

^a^ Percentage is used for categorical variables. Continuous variables are described by their Mean and (Std. Dev.).

^b^ HAZ means height-for-age z score.

^c^ Maternal illiterate and paternal illiterate are both defined as no formal years of schooling attained.

*P*<0.05, **P<0.01, ***P<0.001; Chi-square or ANOVA tests are used to compare child nutritional status and sociodemographic characteristics for categorical and continuous variables across place of residence, respectively.

Child HAZ for mountain areas subsample was significantly lower than that of their counterparts (*F* = 8.81, *P*<0.001). The prevalence of stunting among children in mountain areas is 16.06%, higher than those in plain areas (10.66%) and in the edge areas (8.19%) respectively. Strong differences in the level of the socioeconomic determinants with the exception of household income and household size were found in mountain areas compared with their counterparts. The level of maternal education was especially lower in mountain areas (*F* = 19.44, *P*<0.001); about 60% of mothers attained no years of schooling (*χ^2^* = 50.24, *P*<0.001) in mountain areas while only 40% of mothers shared this strait in plain areas or in the edge areas. The level of paternal education is similarly lower in mountain areas; with an illiterate prevalence nearly double that for the edge areas. The use of piped water was lower in mountain areas but the magnitude of difference is low. The time access to the nearest health clinics was poorer for mountain households as well. Additionally, the proportion of Hui minorities was higher in mountain areas (68%) than in the edge or plain areas (50%).

### Multivariate mixed linear models predicting child HAZ

Table 2 presents the multivariate mixed linear models predicting child HAZ. After adjusting for demographic characteristics (Model 1), coefficients for place of residence on child HAZ was still significant (plain versus mountain, *β* = 0.16, *P* = 0.033; edge versus mountain, *β* = 0.29, *P* = 0.002). Child HAZ was significantly higher in the Han ethnicity group than in the Hui minorities (Han versus Hui, *β* = 0.29, *P* = 0.000), but there was no gender difference (boy versus girl, *β* = 0.07, *P* = 0.235). A significant decreasing trend in child HAZ with increasing age was also observed.

**Table 2:** Multivariate mixed linear regression models predicting child HAZ^a^.

	Model 1	Model 2	Model 3
Fixed effects parameters	*β*(95%CI) b	*β* (95%CI) b	*β* (95%CI) b
Intercept	–0.14 (–0.48, 0.20)	–1.46 (–2.37, –0.55)**	–1.43 (–2.35, –0.52)**
Place of residence: (ref. = mountain areas)			
Plain areas	0.16 (0.01, 0.31)*	0.12 (–0.04, 0.27)	–0.03 (–0.22, 0.16)
Edge areas	0.29 (0.11, 0.48)**	0.25 (0.06, 0.44) **	0.04 (–0.21, 0.29)
Child age	–0.09 (–0.12, –0.06)***	–0.11 (–0.14, –0.07)***	–0.11 (–0.14, –0.07)***
Child gender: boys (ref. = girls)	0.07 (–0.05, 0.19)	0.09 (–0.03, 0.21)	0.09 (–0.03, 0.21)
Child ethnicity: Han (ref. = Hui)	0.29 (0.17, 0.42)***	0.21 (0.07, 0.35)**	0.18 (0.04, 0.33)*
Maternal education	NA^c^	0.01 (–0.01, 0.03)	–0.03 (–0.07, 0.00)
Paternal education	NA^c^	0.01 (–0.01, 0.03)	0.01 (–0.01, 0.03)
Log household income per capita	NA^c^	0.18 (0.01, 0.34)*	0.18 (0.02, 0.35)*
Household size	NA^c^	0.01 (–0.04, 0.06)	0.01 (–0.04, 0.06)
Maternal age	NA^c^	0.02 (0.01, 0.04)**	0.02 (0.01, 0.04)**
Drinking water: tap water (ref. = non-tap water)	NA^c^	–0.03 (–0.19, 0.12)	–0.04 (–0.19, 0.12)
Log time access to nearest clinics	NA^c^	0.06 (–0.11, 0.24)	0.06 (–0.11, 0.23)
Maternal education by place of residence			
Maternal education*plain vs. mountain	NA^c^	NA^c^	0.06 (0.01, 0.10)**
Maternal education*edge vs. mountain	NA^c^	NA^c^	0.07 (0.02, 0.12)**
Log likelihood	–2291.57	–2155.62	–2150.78
**Random effects parameters**			
Between individual variance (95% CI)	1.14 (0.99, 1.30)	1.13 (0.98, 1.29)	1.12 (0.98, 1.29)
Between household variance (95% CI)	0.21 (0.11, 0.41)	0.22 (0.11, 0.42)	0.21 (0.11, 0.41)

^a^ HAZ means height-for-age z score.

^b^ *β* means regression coefficient; CI means confidence interval.

^c^ NA means not applicable.

^*^ *P*<0.05, **P<0.01, ***P<0.001.

With further addition of socioeconomic composition including parental education, household income, resource of drinking water, time access to health clinics and as well as maternal age and household size (Model 2), coefficients for place of residence on child HAZ were decreased in magnitude but remain significant (plain versus mountain, *β* = 0.12, *P* = 0.137; edge versus mountain, *β* = 0.25, *P* = 0.009). Maternal education was not significantly associated with child HAZ (*β* = 0.01, *P* = 0.308). Household income and maternal age were also good predictors of child HAZ, with better nutritional status among children with older mothers and in richer households. Significant associations between other socioeconomic factors and child HAZ were not observed.

**Figure 1:**
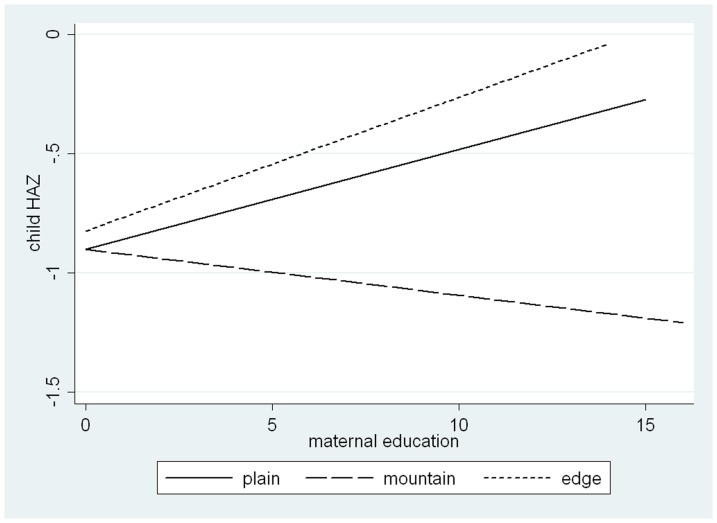
Relationship between maternal education and child HAZ, by place of residence (mountain, plain and edge).

Model 3 and Figure 1 show the interaction between maternal education and place of residence on child HAZ after adjusting for all other covariates. On one hand, as shown in Figure 1, when year of schooling mothers achieved equals 0, the regression lines for plain and mountain areas crossed and they almost crossed with that for edge areas, indicating that for mothers who had never attended school better residential place of plain or edge areas does not efficiently improve child nutritional status. This was also proven by the coefficients for place of residence in Model 3 (plain versus mountain: *β* = –0.03, *P* = 0.778; edge versus mountain: *β* = 0.04, *P* = 0.743), indicating that there is no micro-geographic disparities in nutritional status among children born to mothers who had never attained formal education. Post-hoc estimation using *Wald z* tests showed that until year of schooling mothers achieved increased to 4 years, significant micro-geographic disparities were observed (plain versus mountain: *β* = 0.19, *P* = 0.019; edge versus mountain: *β* = 0.26, *P* = 0.048). However, Figure 1 showed that the disparities between mountain areas and other areas of plain or edge increased with increasing years of schooling mothers attained respectively; as indicated by the significant product terms of maternal education and place of residence in Model 3 (maternal education*plain versus mountain: *β* = 0.06, *P* = 0.007; maternal education*edge versus mountain: *β* = 0.07, *P* = 0.005), these disparities were significant. The gaps of HAZ in children in mountain areas compared with in plain areas and the edge areas significantly widened by 0.06 and 0.07 with an additional year of maternal schooling, respectively.

On the other hand, as shown in Figure 1, the effects of maternal education did vary by different micro-geographic locations. This is also indicated by the significant coefficients for interaction term in Model 3 (maternal education*plain versus mountain: *β* = 0.06, *P* = 0.007; maternal education*edge versus mountain: *β* = 0.07, *P* = 0.005). The regression line of child HAZ for mountain areas decreased as maternal education increased, as indicated by the coefficient for maternal education (separate/main effect of maternal education) in Model 3 (*β* = –0.03, *P* = 0.056), while those for plain and edge areas increased, as shown in post-hoc estimation using *Wald z* tests (plain areas: *β* = 0.02, *P* = 0.094; edge areas: *β* = 0.04, *P* = 0.055).

## Discussion

Prevalence of stunting among rural school-aged children in Northwestern China with low- and middle-SES was about 16% in 2005 [34]. Significant improvement in child nutritional status was observed in the present study with stunting prevalence of 11.68%. Malnutrition was more prevalent among children with older age and the Hui minority while gender was not associated with malnutrition, consistent with previous studies [35]. Moreover, there still existed micro-geographic disparities in the minority area, with worse nutritional status for children in mountain areas, even after adjusting for demographic characteristics (age, gender and ethnicity) of children. Marked differences by place of residence were found in the levels of socioeconomic determinants examined. The mountain areas generally showed more unfavorable parental education, and, to a lesser extent, accessibility of water and health clinics, and ethnic characteristics. Mothers and fathers living in mountain areas were about one and an half to two times more likely to have never attended school compared with those living in plain and in the edge areas, respectively. Consistent with the previous research on geographic disparities in child malnutrition between urban and rural areas, and between Northern regions with dry climate and other regions within Cameroon [7], [13], these findings suggest that the relative advantage in socioeconomic composition of plain and the edge areas over mountain areas may partially mediate the micro-geographic disparities in children’s nutrition. Such an advantage could promote children’s nutrition not only by subsidy of public and health goods and services beneficial to child nutrition but also by better caring practices for children that reflect a parent’s health knowledge, allocation of family resources, and health decision-making power [22], [36]. The micro-geographic disparities in nutritional status declined after further adjusting for socioeconomic factors, but persisted, which may be attributed to some unmeasured contextual factors such as quality of care and climate (e.g. poorer quality of care, less arable land, and more drought in mountain areas) [13], [37].

However, micro-geographic disparities in child nutritional status in the study depended on or were modified by the level of maternal formal education. When maternal education equaled 0 year, there were no significant disparities by place of residence. Until the length of schooling achieved by mothers increased to 4 years, such significant disparities were observed. Moreover, the disparities in mountain areas compared with plain areas and the edge areas respectively widened with increasing years of mothers’ schooling. These findings indicate that access to and use of the favorable resources in plain areas and the edge areas require higher level of knowledge and skills, such as better using of health facilities, interacting effectively with health care providers, and complying with treatment recommendations. In other words, better residential resources more efficiently benefit children with more educated mothers. Vulnerable children with less educated mothers would therefore lose opportunities to enjoy public and health care [27], [30]. The disadvantaged households thus get caught in a vicious spiral. Therefore, these findings emphasize the importance of completing as much formal education as possible and eliminating structural barriers (e.g. complicated terms used in provider-patients communication) simultaneously. Additionally, more targeted and efficient efforts should be taken to reach the vulnerable groups who not only have mothers with low educational attainment but also live in areas with unfavorable socioeconomic and environmental conditions to bridge the unmet maternal educational demands of health status for children.

Our data also found that the main effect of maternal education on child nutritional status showed no significance for the whole sample and corroborated the hypothesis that its effect varied by place of residence, with a negative slope for mountain areas but a positive slope for both plain areas and the edge areas. Moreover, the difference in the strength of the effect was significant in mountain areas compared with plain and the edge settings, respectively; mothers’ education mattered more in plain areas and in the edge areas. There are three possible reasons for geographic patterns in the effect of maternal education. First, there exist some nonlinearities and threshold in the relationship between maternal education and child health [38]. A Ghanaian study found a negative effect at lower levels of schooling on child HAZ but a positive effect at higher education levels with the quadratic specification used [25]. Another Nigerian study showed that there was hardly any significant effect of mother’s education on child HAZ if mothers did not go past primary education [38]. It also corroborated that there was a threshold at five years of mother’s schooling [39] and argued that low cognitive ability through lower education, low quality of overall education, and ineffective health education in curricula gave rise to a fixed cost, and thus to the threshold in mother’s education-child health relationship [38]. Therefore, mothers’ very low level of schooling (only 3.37 years and 2.39 years on average for the whole sample and in mountain areas, respectively), lower than that of Chinese rural women aged 30 through 49 years old in 2000 (5.52 years) [40], would require a very large investment in female education to reach the threshold that could protect child health. Second, more educated mothers may be more likely employed in the market and thus reduce time of child care. Some more educated mothers in rural mountain areas had to leave their poor homeland and rush into cities as migrant workers for more income while their children were left at homeland. However, the negative effect of reduced time on child health couldn’t be offset or traded off by the positive effect of increased income from employment [22], [41]. Third, the effect of maternal education is constrained by an exogenous environmental prerequisite that could vary by place of residence as discussed above, for example, it depends on the minimum access to and quality of care [42]. The increased journey time could be a plausible reason for reduced nutritional status when seeking care for such conditions as children’s diarrhea or maternal delivery occurs in the minority area, especially mountain areas. A lack of drugs and sterilized equipment, dysfunctional staff configurations, inadequately skilled staff and poor infrastructure were also identified as barriers to quality care of village clinics in Ningxia Hui Autonomous Region, which was particular true in Guyuan, the surveyed area in the study [43]. Such unfavorable conditions in residence of mountain areas thus may aggravate the negative effects of low maternal education. It can therefore be inferred that only increasing human capital investment in female education may not be sufficient and innovative interventions on structural differences by place of residence can make such investment more effective.

However, it is also important to recognize that plain or edge areas with favorable conditions are not uniform and that simple micro-geographic comparisons are misleading because they mask the enormous educational differentials in child nutritional status found within plain or edge areas. This is different from the previous studies that have shown mothers’ education mattered more in rural areas or other geographic locations with unfavorable conditions, where efficient use of resources more depends on mothers’ efforts [42]. The fact that differences in socioeconomic and ecological environments at the broad geographic level are different from those at the local level may explain the different geographic patterns in the effect of maternal education.

Policies and programs must be developed with the specific characteristics of the targeted population. However, the micro-geographical pattern is similar to previous studies that have showed larger socioeconomic (indicated by SES index based on household assets, housing quality and availability of services) differentials in child stunting in urban areas than in rural areas [8]. There are distinct groups of children in plain or edge areas who live in as precarious conditions as their counterparts in mountain areas and who are as vulnerable and at-risk of poor nutrition as mountainous areas children. Thus, malnutrition in plain and in edge areas continues to be of concern, and effective targeting of nutrition programs to the least educated groups in plain and in edge areas will be critical to their cost-effectiveness. While these results demonstrate micro-geographical disparities specifically within a minority area in China, they are likely to be relevant to international populations as well. For example, in Cameroon, children living in the driest regions (northern regions) had worse HAZ compared with other regions [13].

The study has several limitations. First, the study was cross-sectional in design and hence the analysis did not permit causal inferences among maternal education, place of residence and child nutritional status. Second, while we recognize that genetic factors and child height matter, unfortunately the data does not permit the inclusion of them which are commonly unobserved by researchers [22]. Nevertheless, it does appear that the effect of maternal education varies by place of residence and micro-geographic disparities widen with increasing levels of maternal education, which has important policy implications. The final limitation is that the results may not be generalized to all poor minority areas in China because there are many socioeconomic and cultural ecological disparities among them.

Despite these limitations, our study still provides some valuable insights into mothers’ educational differentials in child nutritional status by micro-geographic locations in a poor minority area of China, which provides new evidence from Chinese cultural setting to international literature that demonstrate socioeconomic differentials and geographic disparities in health.

## Conclusions

While child nutritional status has generally improved in the Chinese poor minority area, micro-geographic disparities still exist, with worse nutritional status in mountain areas. The micro-geographic disparities widened with increasing education mothers achieved. The association of maternal education with child nutritional status may vary by micro-geographic locations, with a negative association in mountain areas and positive associations in plain and in edge areas, respectively. Thus, enlarging female education alone is not sufficient; improving unfavorable conditions in mountain areas can make such investments more effective in promoting child health. Moreover, malnutrition in plain and in edge areas continues to be of concern, and effective targeting of nutrition programs to the least educated groups in plain and in edge areas will be critical to their cost-effectiveness.
